# Preserving the Integrity of Liposomes Prepared by Ethanol Injection upon Freeze-Drying: Insights from Combined Molecular Dynamics Simulations and Experimental Data

**DOI:** 10.3390/pharmaceutics12060530

**Published:** 2020-06-09

**Authors:** Silvia Franzè, Francesca Selmin, Paolo Rocco, Giuseppe Colombo, Antonella Casiraghi, Francesco Cilurzo

**Affiliations:** 1 Department of Pharmaceutical Sciences, University of Milan, Via G. Colombo 71, 20133 Milan, Italy; silvia.franze@unimi.it (S.F.); paolo.rocco@unimi.it (P.R.); antonella.casiraghi@unimi.it (A.C.); francesco.cilurzo@unimi.it (F.C.); 2 Italfarmaco S.p.A., Viale Fulvio Testi 330, 20126 Milan, Italy; giuseppe.colombo@italfarmaco.com

**Keywords:** cake appearance, cryoprotectant, freeze-drying, freezing rate, liposomes, lyoprotectant, solvent, sublimation, time and temperature

## Abstract

The freeze-drying of complex formulations, such as liposomes, is challenging, particularly if dispersions contain residual organic solvents. This work aimed to investigate the effects of possible protectants, namely sucrose, trehalose and/or poly(vinyl pyrrolidone) (PVP), on the main features of the dried product using a 1,2-dipalmitoyl-sn-glycero-3-phosphocholine (DPPC)-based liposomal dispersion prepared by ethanol injection and containing ethanol up to 6%, as a model. The interactions among vesicles and protectants were preliminary screened by Molecular Dynamics (MD) simulations, which have been proved useful in rationalizing the selection of protectant(s). The freeze-drying protocol was based on calorimetric results. Overall data suggested a stronger cryo-protectant effect of trehalose, compared with sucrose, due to stronger interactions with the DPPC bilayer and the formation of highly ordered clusters around the lipids. The effect further improved in the presence of PVP. Differently from the other tested protectants, the selected trehalose/PVP combination allows to preserve liposome size, even in the presence of 6% ethanol, as demonstrated by Nanoparticle Tracking Analysis (NTA). Nevertheless, it should be also underlined that cakes blew out at an ethanol concentration higher than 1% *v*/*v*, probably due to the poor cohesion within the cake and solvent vapour pressure upon sublimation.

## 1. Introduction

Being among the most versatile carriers to effectively deliver active substances, liposomes have been investigated for a wide range of applications in the food, cosmetic and pharmaceutic industries. Due to the increasing interest, preparation methods have been evolving from conventional laboratory scale techniques based on top-down approaches which require post-processing steps (e.g., thin film hydration method) to bottom-up technologies enabling to tailor liposome size with a good reproducibility and scalability. Among them, the ethanol injection method allows to achieve a good control over the physical properties of liposomes and can be scaled-up at the industrial level [1]. However, the elimination of residual ethanol may be problematic because of the formation of an azeotropic ethanol/water mixture [2]. Lyophilization has the potential to yield a dried product in a single operation with the concomitant advantages of facilitating transportation, storage and prolonging shelf-life. However, freeze-drying can cause structural changes to liposomes as a result of freezing and dehydration stresses: ice crystals can damage bilayer membranes through mechanical stress, whereas water removal can induce fusion and/or aggregation phenomena [3]. Therefore, formulation design and lyo-cycle conditions should be specifically studied. In general, adding cryo-protectants and/or lyo-protectants leads to effective preservation of the liposomes and allows easy reconstitution of the lyo-cake. According to literature data, the choice of excipients suitable for liposome lyophilization is limited mainly to monosaccharides and disaccharides [3,4], which can either replace water molecules in stable hydrogen bonds with lipid head groups and/or form a glassy matrix spacing between adjacent lipid bilayers, thus avoiding fusion and mechanical disruption. In order to obtain a uniform lyo-cake, cryo- and/or lyo-protectants may be combined to other excipients acting as bulking agents, which are necessary to assure a suitable physical structure. Excipients are usually selected on calorimetric studies focused on the thermotropic pattern of phospholipids in their presence. Conversely, few examples of in silico rationalization of such excipient are available in the literature [5,6,7]. Since formulation and freezing parameters determine the specific surface area of the lyo-cake [8,9,10], the design of appropriate freezing processes (i.e., temperature and rate) can also improve the quality of the final product in terms of primary drying performance [10,11], desorption rate during the secondary drying [12] and reconstitution time [13]. The presence of ethanol raises some concerns at the formulation selection and process optimization stage due to its influence on freezing and sublimation steps [14]. As an example, ethanol can retain significant amounts of associated water molecules which only partially freeze as the temperature decreases [15]. Moreover, unfrozen aliquots of ethanol may boil during the early primary drying phase, causing defects in the matrices [16], long process times and heterogeneous residual solvent levels in final products [14]. However, to the best of our knowledge, literature reports focus on biotherapeutics and very few data are available on freeze-drying liposomal dispersion in the presence of organic solvents [3].

Here, we aimed to explore two aspects related to the freeze-drying of a liposomal dispersion: the first is to assess the usefulness of Molecular Dynamics (MD) simulations into predicting the protective effect of auxiliary excipients; the second is to study the feasibility to freeze-drying formulations in the presence of ethanol.

A liposomal dispersion made of 1,2-dipalmitoyl-sn-glycero-3-phosphocholine (DPPC) and cholesterol (CHOL) (70:30, mol:mol ratio) was prepared by the ethanol-injection method. This placebo and basic composition was used as a model to easily assess the thermotropic phase behavior of phospholipids in the presence of protectants. Moreover, DPPC and CHOL bilayers are stable against the ethanol fluidifying effect that may favor the premature vesicle fusion in the time gap between liposome preparation and lyophilization. On the basis of a preliminary freeze-thawing screening, trehalose and sucrose were selected as protectants while poly(vinyl pyrrolidone) (PVP) was chosen as a bulking agent [17].

## 2. Materials and Methods

### 2.1. Materials

1,2-dipalmitoyl-sn-glycero-3-phosphocholine (DPPC) was kindly provided by Lipoid Gmhb (Ludwigshafen, Germany). Cholesterol (CHOL) was purchased from Sigma-Aldrich (Milan, Italy). Trehalose was purchased from AppliChem GmbH (Darmstadt, Germany). Sucrose was obtained from Farmalabor (Canosa di Puglia, Italy). Poly(vinyl pyrrolidone) K12 (PVP) was a gift from BASF (Ludwigshafen am Rhein, Germany). All solvents were of analytical grade, unless specified.

### 2.2. Molecular Dynamics Simulations

**Figure 1:**
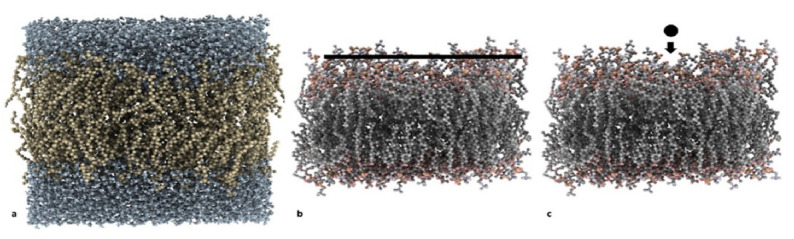
(**a**) Hydrated 1,2-dipalmitoyl-sn-glycero-3-phosphocholine (DPPC):cholesterol (CHOL) bilayer used in phase I and phase II Steered Molecular Dynamics (SMD) set 1 simulations; (**b**) setup for phase I simulations (the disaccharide layer is shown as a black line, and the water molecule is not shown); and (**c**) setup for phase II SMD set 1 simulation (the black dot represents the disaccharide pulled from bulk water into the bilayer, and the water molecule is not shown).

A theoretical 70:30 DPPC:CHOL model bilayer was generated starting from a previously prepared DPPC bilayer equilibrated through a 100-ns MD simulation at 300 K at the constant pressure of 1 atm (NPT). In the DPPC bilayer, 30% of the DPPC molecules were randomly replaced with CHOL molecules. The obtained bilayer was then equilibrated through a 50-ns constant temperature and volume (NVT) MD simulation at 300 K. All molecules were in neutral form. The final bilayer consists of 140 DPPC molecules, 60 CHOL molecules and two water layers, each containing about 3159 water molecules (Figure 1a) [18]. The DPPC:CHOL bilayer was used in three sets of MD and Steered MD (SMD) simulations:

1. MD set: MD simulations, where a layer of 48 sugar (e.g., sucrose or trehalose) molecules was placed on top of the membrane and the change in its physicochemical properties during the simulation was analyzed (Figure 1b);
2. SMD set 1: SMD simulations, where a single sugar molecule was pulled from bulk water into the bilayer (Figure 1c); and
3. SMD set 2: SMD simulations, where DPPC molecules were pulled from a PVP/trehalose cluster or from a PVP layer.

In both cases, simulations were performed at constant temperature and volume (NVT) with the following characteristics:

- Periodic boundary conditions were applied to stabilize the simulation space.
- Newton’s equation was integrated using the r-RESPA method (every 4 fs for long-range electrostatic forces, 2 fs for short-range non-bonded forces, and 1 fs for bonded forces).
- The long-range electrostatic potential was computed by the Particle Mesh Ewald summation method, and the chosen cutoff length was 12 Å for both the Van der Waals and electrostatic potentials, with a switching function starting at 8 Å.
- The temperature was maintained at 300 ± 10 K by Langevin’s algorithm.
- Lennard–Jones (L.-J.) interactions were calculated with a cutoff of 10 Å, and the pair list was updated every 20 iterations.
- A frame was memorized every 1 ps.
- No constraints were imposed to the systems.

Simulations were performed using NAMD 2.10 [19] (University of Illinois at Urbana-Champaign, Champaign, IL, USA) with CUDA^®^ (Nvidia Corporation, Santa Clara, CA, USA) support on a Microsoft Windows^®^ PC assembled with off-the-shelf components. The force fields used are CHARMM 36 for the lipids, the disaccharides and poly(vinyl pyrrolidone) (PVP) and are TIP3P for water [20,21].Trajectory analysis and calculation of physicochemical properties were performed in VEGA ZZ [22].

### 2.3. Liposome Preparation

#### 2.3.1. Ethanol Injection

The experimental variables considered to obtain liposomes of about 150–180 nm are listed in the Supplementary Materials (Table S1). Briefly, DPPC and CHOL in the molar ratio of 70:30 (total concentration fixed at 25 mM) were dissolved in absolute ethanol. The system was heated at 55 °C (above the gel-to-liquid crystal phase transition temperature of DPPC) to improve the miscibility of the phospholipid into the organic solvent. Then, 1 mL of ethanolic solution was loaded in a 21-gauge syringe and injected at the rate of 1 mL/min in 16 mL of MilliQ^®^ water, kept at 55 °C and magnetically stirred at 300 rpm. The rate of injection was maintained constant by pushing the syringe plunger with a probe of an electronic dynamometer (INSTRON^®^ 5965, ITW Test and Measurement Italia S.r.l, Pianezza, Italy). Spontaneous formation of unilamellar liposomes occurred as soon as the organic phase was in contact with the aqueous phase. Then, the liposomal dispersion was kept for 30 min under stirring at room temperature. The final ethanol concentration in the raw liposomal dispersion was 6% *v*/*v*. To obtain dispersions with lower ethanol content, namely 0.1% and 1% *v*/*v*, instead after liposome preparation, the residual solvent was partially removed by rotary evaporation (RII, Buchi, Cornaredo, Italy) or under constant flux of nitrogen, respectively.

#### 2.3.2. Thin-Film Hydration Method

Ethanol-free liposomes were prepared using the thin-film hydration method. Briefly, DPPC and CHOL were dissolved in chloroform and put into a round flask. The organic solvent was evaporated under reduced pressure (80 mbar) at 50 °C and 100 rpm for 1 h using a rotatory evaporator (RII, Buchi). The lipid film was rehydrated with MilliQ^®^ water for 1 h at 50 °C, leading to the formation of multilamellar vesicles. Liposomes were extruded then 6 times through 0.1-μm polycarbonate membranes (Nucleopore Track-Etch Membrane, Whatman^®^, Maidstone, UK) to obtain unilamellar vesicles. The reproducibility data of size, polydispersity index (PDI) and ζ-potential values of liposomes prepared by both methods are listed in Table S2. Liposomal dispersions were stored at 4 °C until use.

### 2.4. Liposome Characterization

The particle size distributions of liposomes before freeze-dying and after reconstitution were assessed both by dynamic light scattering (DLS) and Nanoparticle Tracking Analysis (NTA). For DLS, samples diluted 1:10 with ultrapure water (refractive index: 1.345; absorption: 0.010) were analyzed at 25 °C with a scattering angle of 173° using a Zetasizer (Nano-ZS, Malvern Instrument, Worcestershire, UK). All the results are expressed on an average of three measurements, calculated on average of 11 runs. NTA analyses were carried out using a Nanosight NS300 (Malvern Instrument, Malvern, Worcestershire, UK) after 1:100,000 sample dilution with ultrapure water filtered with 0.2-μm nylon filter. Analyses were performed at 25 °C, and results are expressed as the mean of three measurements. ζ-potential of liposomes was assessed on the samples diluted 1:10 by using the Zetasizer. The results are expressed as the mean of three determination and standard deviation.

### 2.5. Residual Ethanol Content

The residual ethanol content in liposome prepared by ethanol injection was determined by gas chromatography using a Trace GC (Thermo Electron, Rodano, Italy) equipped with a flame ionization detector (FID) and an VF-624 ms fused silica capillary column (30 m length, 0.25 mm I.D., 0.25 μm film thickness; Varian, Segrate, Italy). The carrier gas was helium (1.3 mL/min volumetric flow). The injector and detector temperature were 150 and 250 °C, respectively. The temperature ramped from 50 to 150 °C at a rate of 10 °C/min. Quantitative data were obtained by using n-propranolol as inner standard.

### 2.6. Compatibility Study

To test experimentally the compatibility between liposomes and the selected excipients, trehalose or sucrose were added in 5:1 molar ratio with respect to DPPC. The mixture was incubated at 25 ± 1 °C for 1.5 h under stirring at 100 rpm in a benchtop incubation shaker (Sartorius Certomat IS, Varedo, Italy). When appropriate, an aliquot of PVP solution was added and samples were shaken further for 30 min under the same conditions. Since the presence of visible aggregates was considered an index of incompatibility among constituents, only clear dispersions were subjected to DLS.

### 2.7. Freeze-Thawing

To assess the cryoprotective role of excipients, 1 mL liposomal samples were frozen at −40 °C at the freezing rate of 1 K min^−1^, kept at −40 °C for 30 min and thawed at 1 K min^−1^ to 20 °C in a freeze-drier (Epsilon 2–6D LSCplus, Martin Christ, Osterode am Harz, Germany). After thawing, only clear dispersions were subjected to DLS analysis.

### 2.8. Evaluation of the Fluidity of the Membrane in the Presence of Ethanol

Liposomal dispersions containing 1% and 6% *v*/*v* of residual ethanol were loaded in a gas-tight syringe, for which the plunger was put in contact with a 50-N load cell of a dynamometer (INSTRON 5965, ITW Test and Measurement Italia Srl, Pianezza, Italy) and pushed at a constant speed of 1 mm/s, forcing the liposomal dispersion through a 50-nm polycarbonate membrane fixed in an extruder casing. The force (N) required to move the syringe plunger (dependent on the resistance opposed to vesicle penetration through the pores) was registered as a function of the plunger displacement (mm). The slope of this plot, namely the constant of deformability (k), was then derived. For k value approaching zero, the membrane is fluid and the quick rearrangement of the vesicles in the pores occurs [23].

### 2.9. Thermal Study on DPPC-Excipient Interactions

Lipid phase behavior and phase transitions were monitored by Differential Scanning Calorimetry (DSC, DSC1 Stare System, Mettler Toledo, Milano, Italy) on DPPC multilamellar vesicles prepared by the thin-film hydration method. The use of cholesterol was avoided since it is known to significantly decrease the enthalpy variation associated with the solid-to-liquid state transition of the phospholipid hydrocarbon chains and thereby jeopardizes DSC investigations [24]. To assess also the possible interaction between phospholipid head groups and protectant(s), DPPC films were hydrated with (i) pure water, in which, afterwards, the solution of protectants was added to lamellar phase samples, or (ii) directly with the solution containing the selected excipient(s).

An aliquot of about 30 µL exactly weighed was transferred to an aluminum pan, sealed and subjected to cooling until 0 °C at 1 K min^−1^, kept at 0 °C for 5 min and then heated to 60 °C at 2 K min^−1^. The DSC cell and refrigerated cooling systems (RCS) were purged with dry nitrogen at 80 and 120 mL/min, respectively. The system was calibrated using an indium standard. All data were treated with Star^e^ System software Version 10.0 (Mettler Toledo).

### 2.10. Freeze-Drying

To tailor the process parameters of freeze-drying [25], the glass transition temperature of a maximally cryo-concentrated solution (T_g_′) of trehalose and PVP solution both in aqueous and hydro-alcoholic solutions were determined. Briefly, an aliquot was cooled until −40 °C at 5 K min^−1^, kept at −40 °C for 20 min and then heated to 25 °C at 5 K min^−1^ [26]. T_g_′ was taken as the inflection point of the specific heat increment at the glass–rubber transition on the heat scan.

Then, vials (R2, Schott, Mainz, Germany) were filled with 0.9 mL of the product solution, semi-stoppered, and placed on shelves in hexagonal packaging. Product temperature was monitored for 3 center vials and 3 edge vials via Wi-Fi and wire thin thermocouples, respectively. A stainless-steel tray bottom with a stainless-steel frame was used to transfer the vials into the freeze-dryer, and the tray bottom was removed prior to freeze-drying.

The samples were freeze-dried using an Epsilon 2–6 LSC plus freeze dryer (Martin Christ) according to three different processes. Briefly, depending on the T_g_′ value, the samples were frozen at the rate of 0.5 K min^−1^ to a minimum shelf temperature of −40 °C or −48 °C and held for 8 h to ensure complete freezing. Two different temperatures were set in the primary drying. Samples containing 0.1% ethanol concentration were kept at −40 °C and 0.2 mbar for 48 h (method A, Table 1). Afterwards, the shelf temperature was increased to 25 °C at the rate of 1 K min^−1^ to initiate the secondary drying. The desorption phase was carried out over a 10-h period. In case of samples at the highest ethanol content (i.e., 1% and 6%), the sublimation was carried out at −48 and −40 °C for 12 and 13 h, respectively (method B, Table 1). For the secondary drying, the temperature was increased to 25 °C at 0.1 K min^−1^ and held for 6 h. In both phases, the chamber pressure was 0.1 mbar. Finally, method B was further modified, splitting the primary drying into two segments at −48 and −40 °C, respectively, and reducing the pressure at 0.1 mbar (method C, Table 1).

### 2.11. Residual Water Content

Residual moisture was measured by a Volumetric Karl Fischer titration V20 (Mettler Toledo, Milano, Italy). A volume of 0.6 mL anhydrous methanol was added, and vials were then intermittently shaken for 1 h. Afterwards, samples of 300 μL of methanol were injected into the titration vessel. The water content was measured in triplicate.

### 2.12. Reconstitution

Reconstitution is one of the less standardized procedures, despite its importance as a key determinant of product quality. However, the act of reconstitution, although usually performed by trained staff, is nevertheless impacted by the “human factor”. In an attempt to avoid the subjectivity of manual reconstitution, an automatic and therefore standardized shaking protocol was defined. Freeze-dried products were reconstituted with 0.9 mL MilliQ^®^ water to obtain the initial concentration and shaken at 100 rpm and 25 °C for 30 min in a benchtop incubation shaker (Sartorius Certomat IS, Varedo, Italy). Under these conditions, lyo-product dissolved in less than 1 min, but further time was required to achieve the complete liposome hydration and the obtainment of a reproducible data in terms of size and DPI.

## 3. Results

### 3.1. Molecular Dynamics Simulations

Three sets of MD simulations were performed as a preliminary investigation of the nature and extent of the interactions between the different compounds involved and as a means of rationalizing the subsequent experimental part. These were as follows:

- a set of MD simulations on system comprised of a layer of disaccharides and a DPPC bilayer to investigate the cryoprotectant effect of the disaccharides (MD set);
- a first set of Steered MD simulations to compare the energy of the trehalose-bilayer and sucrose-bilayer interactions (SMD set 1); and
- a second set of Steered MD simulations to compare the energy of the PVP-bilayer and trehalose-PVP-bilayer interactions (SMD set 2).

**Figure 2:**
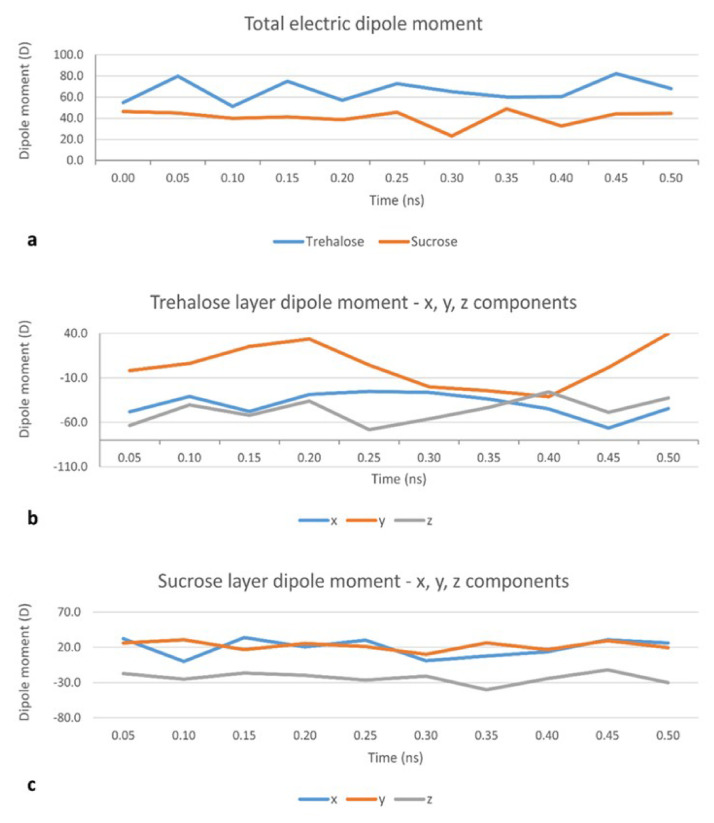
Evolution of total dipole moment of the whole sugar layer during the Molecular Dynamics (MD) set, showing consistently higher values for the trehalose layer (**a**): Evolution of the x, y and z components of the dipole moment (µx, µy and µz, respectively) for the trehalose layer (**b**) and the sucrose layer (**c**), showing a higher absolute value and higher weight with respect to the x and y components, of µz for trehalose than for sucrose; z is the direction of the bilayer’s normal, and xy is the plane of the bilayer’s surface.

In the MD set, a layer of 48 disaccharide molecules was generated in VEGA ZZ with random rotations about the three spatial axes. The center of mass of the obtained sugar layer was placed on top of the membrane at a distance (d) of 20 Å from the bilayer midplane. After minimization, the simulations were carried out in two phases: an initial period of heating from 0 K to 300 K over 30,000 iterations (30 ps, i.e., 10 K/ps) and the monitored phase of 0.5 ns, after which the disaccharide layer starts disrupting. MD simulations from this set were used to obtain a collection of conformations of the disaccharide cluster which are more likely to occur when it interacts with a DPPC:CHOL bilayer. 11 such conformations, once every 50 frames (i.e., 0.05 ns), were further minimized with MOPAC 2016 (James J. P. Stewart, Stewart Computational Chemistry, Colorado Springs, CO, USA), using a PM6 Hamiltonian [27] and their total electric dipole moment calculated. The results are shown in Figure 2a. The total dipole moment for the trehalose layer is consistently higher (60.0 D on average) than the dipole moment for the sucrose layer (48.9 D on average).

The same procedure was used to calculate the electric dipole moment of a single molecule of sucrose (1.44 D in a vacuum and 1.03 D in water) or trehalose (0.50 D in a vacuum and 1.40 D in water). This increase in the electric dipole moment is in line with results suggesting that trehalose is able to bind, through hydrogen bonds, a large number of water molecules [28]. The same pattern applies when a small cluster of 4 disaccharides is considered. In this case, the electric dipole moment of a cluster of 4 disaccharide molecules in a vacuum is 3.80 D for sucrose and 5.06 D for trehalose. Though, as the average electric dipole moments for any single molecules are comparable (1.68 ± 0.90 D for sucrose and 1.67 ± 0.40 D for trehalose), this seems more attributable to a higher degree of order in the orientation of the dipole moments, the latter result is confirmed by an analysis of the three components—µx, µy and µz—of the total electric dipole moment. Indeed, µz (z being the direction of the bilayer’s normal) for trehalose (Figure 2b) has a higher absolute value and higher weight with respect to the x and y components than for sucrose (Figure 2c). The average µz is −46.6 D for trehalose and −24.6 D for sucrose [28]. This, together with the values of the x and y components (lying in the plane of the bilayer’s surface), translates to a smaller angle between the dipole moment for the trehalose cluster and the bilayer’s normal. Taken together, these results suggest that the cryoprotectant role of trehalose may be attributed to both its well-known ability of forming a high number of hydrogen bonds with water molecules [28] and its ability of forming a highly ordered cluster, which ultimately concur to the disruption of water clusters [29] and which is confirmed by the present calculations also in the proximity of a DPPC:CHOL bilayer.

In SMD set 1, after minimization, the simulations were carried out in two phases: an initial period of heating from 0 K to 300 K over 30,000 iterations (30 ps, i.e., 10 K/ps) and the monitored phase of 0.5 ns. During the monitored phase, the sugar molecule (i.e., trehalose or sucrose), initially placed on top of the bilayer at a distance of 26 Å from the bilayer midplane, was forced to cover a distance of 10 Å at a speed of 0.02 Å/ps toward the bilayer boundary by applying a harmonic constraint force equal to 5 Kcal/mol/Å2.

**Figure 3:**
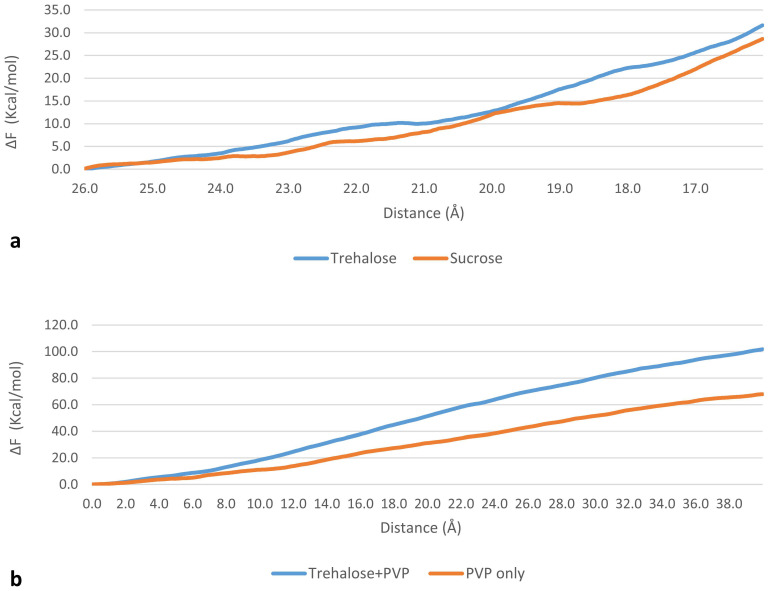
Free-energy difference calculated through Jarzynski equality for (**a**) the process of pulling a sugar molecule from bulk water (d = 26 Å) into the membrane (d < 15.5), showing a higher interaction of trehalose with the bilayer and (**b**) the process of pulling DPPC molecules toward the bilayer’s midplane, showing a stronger interaction of DPPC with a trehalose/poly(vinyl pyrrolidone) (PVP) cluster rather than PVP alone.

The force acting on the sugar molecule was derived directly from the SMD simulations. From that value, the work needed to move a single sugar molecule in each trajectory was calculated and free-energy calculations were performed for both systems (bilayer + trehalose and bilayer + sucrose) using Jarzynski [30] equality:

e^(−∆F/kT)^ = 〈e^(−W/kT)^〉_ens_ (1)

where ∆F is the Helmholtz free-energy difference between the initial and final configurations, W is the work calculated from MD trajectories, the average 〈 〉_ens_ is taken over an ensemble of 10 different trajectories from the same initial conditions, k is Boltzmann’s constant and T is absolute temperature (300 K). A qualitative evaluation of the calculated ∆F, slightly higher for trehalose, suggests a stronger interaction (Figure 3a).

SMD set 2 simulations were performed on a single lipid layer. System A is composed of the DPPC/CHOL monolayer, PVP (center of mass placed at a distance of 21 Å from the bilayer’s midplane) and trehalose layer (center of mass placed at a distance of 20 Å from the bilayer’s midplane), while System B is composed of the DPPC/CHOL monolayer and PVP only. Both systems were equilibrated through a 10-ns MD simulation.

After minimization, simulations were carried out in two phases: an initial period of heating from 0 K to 300 K over 30,000 iterations (30 ps, i.e., 10 K/ps) and the monitored phase of 2 ns. During the monitored phase, all DPPC molecules except for 3 of them were removed. The 3 DPPC molecules were forced to cover a distance of 40 Å at a speed of 0.02 Å/ps toward the bilayer’s midplane by applying a harmonic constraint force equal to 5 kcal/mol/Å2. The SMD simulations were performed on 3 DPPC molecules so that an average effect could be measured.

As in SMD set 1, free-energy calculations were performed for both systems using Jarzynski equality (Figure 3b). Qualitative evaluation of the free-energy difference suggests a stronger interaction of DPPC with the trehalose–PVP cluster than with PVP alone. The calculated ΔF values should not be considered as bond energies, as the simulations were carried over for a much longer time than the time needed for bond breaking. Thus, the trehalose–PVP combination appears more suitable to protect the liposome during the process than the single components. To confirm this hypothesis, the compatibility of the excipients before and after freeze-thawing cycles was determined and the thermotropic behavior of DPPC was assessed.

### 3.2. Liposome Characterization

As well-known from the literature, the main parameters affecting the lamellarity and particle size of liposomes obtained by ethanol injection are the ratio between organic and aqueous phase and the lipid concentration, along with the temperature and injection conditions [31]. Accordingly, the particle size and PdI of prepared liposomes decreased, increasing the volume of aqueous phase and decreasing the lipid concentration (Table S1). In fact, the higher the lipid amount dissolved in the organic phase, the higher the probability to have aggregation at the injection site. Keeping the ethanol content to 6% *v*/*v* and increasing the temperature of the water bath up to 55 °C allowed to obtain unilamellar vesicles with the desired particle size distribution (131 ± 1 nm) and with a good reproducibility (Table S2).

### 3.3. Compatibility and Thawing Study

Since it is well-known that ethanol reduces the Young modulus of the liposome bilayers [32], the contribution of different amounts of ethanol on the fluidity of the membrane, which in turn affects the interaction with the excipients and the stability of the membrane upon process and storage conditions, was evaluated by a dynamometer assisted extrusion assay. As expected, the residual solvent improved the fluidity of the membrane since both the formulations at 1% and 6% residual ethanol presented a k values (k = ~0.003 N/mm) lower than that of formulation prepared by thin-film hydration (k = ~0.04 N/mm). Since the fluidity values of formulations having 1% and 6% *v*/*v* ethanol content were not significantly different, the first one was selected for being representative of the most fluid formulations in further compatibility studies and freeze-thawing experiments.

The addition of disaccharides or their combination with PVP without subsequent freezing had no influence on liposome size. Despite an increase in bilayer fluidity, ethanol improved the stability of liposomes upon a freeze-thawing cycle. Indeed after thawing, no variations in size and polydispersity index (PDI) were measured for liposomes (without excipients added) with an ethanol content ranging from 1% (D_H_ = 142 ± 1 nm; PDI = 0.19 ± 0.0) to 6% *v*/*v* (D_H_ = 151 ± 1 nm; PDI = 0.14 ± 0.0), whereas in the case of vesicles prepared by the thin-film hydration method, DLS analysis did not accomplish the quality criteria due to the presence of too-dispersed particles. Indeed, liposomes frozen without cryoprotectants exhibited an increase in their size combined with a decrease in homogeneity, marked by a remarkable rise in the PDI value. The different behaviors can be attributed to ethanol, which could remain uniformly entrapped as a liquid (T_f_ = −114 °C) in the solid microregions upon freezing [14]. Concomitantly, this space was progressively populated by liposomes which, being dispersed in the solvent, reorganized without undergoing damages caused by freezing or cryo-concentration.

Therefore, to assess the efficiency of protectants, thawing experiments were carried out on liposomes prepared by the thin-film hydration method. The results reported in Table 2 evidenced that trehalose and sucrose at molar ratio 1:5 were both able to preserve liposome size. However, between the selected disaccharides, considerable differences in PdI were observed after thawing when compared to the samples before freezing, and trehalose allowed to maintain the narrowness of liposome population. The addition of PVP was effective to ensure cryoprotection only at 0.5% *v*/*v* and/or in combination with a disaccharide (Table 2). Therefore, it can be concluded that trehalose or a combination of trehalose and low concentrations of PVP is beneficial to maintain homogeneity during freezing.

### 3.4. Thermotropic Behavior of DPPC in Presence of Protectant

Based on MD and thawing data, sucrose was discarded; therefore, the possible interaction with DPPC was evaluated only in the presence of trehalose or the relative mixtures with PVP. According to literature [3], the DPPC lamellar phase in water presented two endothermic transitions at 34 and 41 °C when DPPC multilamellar vesicles underwent gel-to-liquid crystalline transitions (Table 3). The weaker transition or pre-transition at 34 °C (T_p_) represented the transformation from the stable lamellar (Lβ′) to hexagonal ripple (Pβ′) phases and the main transition (T_m_) showed the transformation from Pβ′ to the liquid crystalline (Lα) phase. As described previously, the addition of trehalose as well as PVP to both sides of the bilayers did not modify DPPC pre-transition significantly (one-way ANOVA, *p* = 0.09) [33]. Conversely, trehalose modified the symmetry of the main transition, which was slightly skewed on the low temperature side (Figure S1). This was concomitant to an increase in T_m_ enthalpy (Table 3), suggesting the formation of interactions among molecules of DPPC and trehalose [34], as predicted in the MD simulations.

After the polymer addition, the T_m_ enthalpy massively decreased (Table 3). In general, this variation may indicate the suppression of cohesive interactions between adjacent phospholipid molecules. Regarding DPPC, Savva et al. suggested that not only PVP interacts with polar phospholipid headgroups via H-bonds but also hydrophobic interactions with the phospholipid acyl chains can occur in the bilayer [34]. The slight peak broadening in the presence of PVP can also confirm the increase of membrane heterogeneity that was previously reported in the interactions of other polymers with DPPC bilayer [35].

When DPPC lamellae were hydrated by a solution containing both excipients, the magnitude of enthalpy decrease was lower with respect to PVP and no variation on the onset T_m_ was found (Table 3). Consequently, trehalose mitigates the effect of PVP on the DPPC bilayer. This is confirmed by phase II MD simulations (*SMD set 2*), which suggest that the interaction of DPPC is stronger with a the trehalose–PVP cluster than with PVP alone (Figure 3b).

### 3.5. Impact of Protectants on Freeze-Dried Product Quality

Preliminarily, the thermal behavior of the solutions of trehalose, PVP, and the binary mixture at different ethanol content was elucidated by DSC. In the cosolvent solution of trehalose, the T_g_′ was depressed and the magnitude of such an event was dependent on the residual solvent (Figure S2). In the case of PVP or the PVP mixture at the highest ethanol content, no T_g_′ could be detected in the DSC heating curve (Table 4).

**Figure 4:**
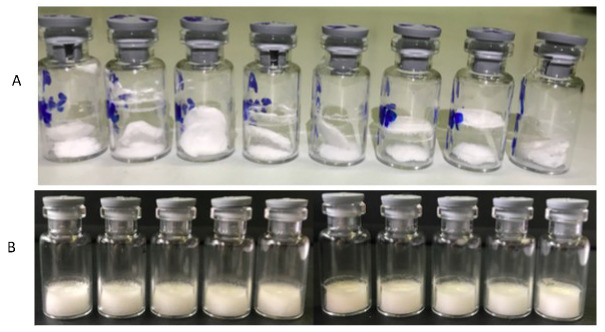
Cakes obtained after lyophilization of formulations with 1% *v*/*v* ethanol content (**A**) and 0.1% *v*/*v* ethanol content (**B**): Figure 4A clearly evidences the phenomenon of “product ejection”.

These results were in agreement with Kunz et al., who reported similar evidence on a solution at 10% ethanol [36]. Even if drying at temperatures above T_g_′ or T_c_ can lead to macroscopic collapse, recent literature data reported that collapse itself does not necessarily affect quality and stability of a product [37,38]. Hence, in a preliminary study, all formulations were subjected to the same freeze-drying cycle (method A, Table 1) and no macroscopic collapse was observed. However, up to 1% ethanol, the finished product “blew out” of the vial (“product ejection”) probably because of poor cohesion within the cake and solvent vapor pressure upon sublimation [16]. In all other cases, the trehalose series presented a good cake appearance and PVP provided macroscopic support, leading to the formation of more “elegant” product cakes (Figure 4).

Thermal behavior of trehalose and before and after freeze-drying was briefly described in the Supplementary Materials (Figure S3).

In the residual moisture content measured by Karl Fischer, titration was in the 2–3% range without significant variations among formulations. Independently of their appearance, these samples were reconstituted in water and the size was evaluated by DLS and NTA analysis if no visible aggregates were present. As expected, after rehydration of freeze-dried liposomes in the absence of protectants, both the size and the PDI were massively increased, suggesting that aggregation and/or fusion occurred during lyophilization. In the presence of protectants, liposome size and PDI strictly depended on the residual ethanol content and the excipient used (Table 5). Interestingly, freeze–drying of liposomes in the presence of trehalose at a 5:1 molar ratio resulted in vesicles sizing up to 500 nm and with a broad PDI upon reconstitution. The addition of 0.5% PVP improved vesicle size retention only at residual ethanol content lower than 1%. Indeed, in this set of samples, drying at a temperature of −40 °C ensured that product temperature was kept safely below T_g_ due to the additional effect of the cooling consequent to sublimation. Samples containing 1% and 6% residual ethanol presented visual aggregates upon reconstitution, and therefore, a different protocol was defined (method B, Table 1).

However, the maintenance of the shelf temperature at −48 °C during the primary drying caused the formation of visible aggregate after reconstitution. To preserve liposome integrity, the sublimation phase was divided into two segments at −48 and −40 °C, respectively (method C, Table 1). Based upon variation of condenser temperature (data not shown), ethanol was mostly removed in the first step. A conservative ramp temperature in the transition to secondary drying was chosen even if the heating rate of 1 K min^−1^ did not cause the cake to collapse. The degree of destabilization due to freeze-drying stress was lower for formulation freeze-dried according to method C (Table 5) compared to other protocols.

**Figure 5:**
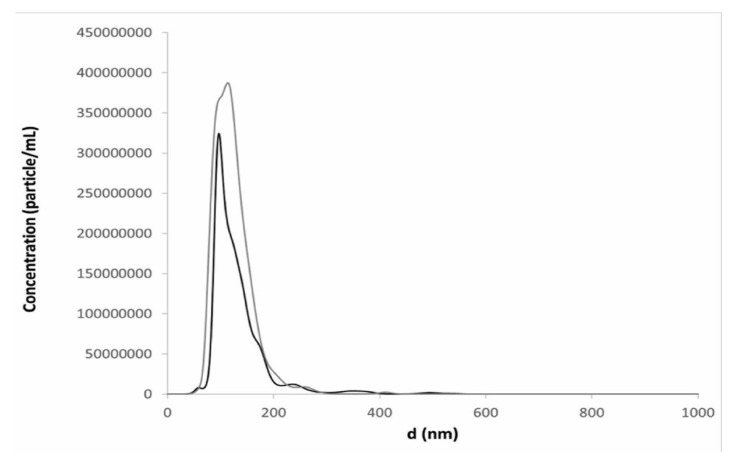
Particle size distribution from Nanoparticle Tracking Analysis (NTA) measurements of resuspended liposomes in the presence of trehalose (black line) and the combination of trehalose and PVP (grey line) after freeze-drying (method B) dispersions with 6% *v*/*v* ethanol content: This profile was obtained by plotting the average data obtained after analyses of 3 reconstituted liposome dispersions.

In fact, the particle size distribution by intensity registered by DLS in a reconstituted sample revealed that most of the liposomes maintained the same dimension and lamellarity as that before freeze-drying (D_H_: 126 ± 6; intensity: 72%). However, the distribution was bimodal with 18% (in intensity) of aggregates with a particle size of almost 1000 nm (Figure S4). Nevertheless, the last population represented less than one million of the main population of vesicles in number distribution, according to NTA data, which showed that liposome structure was maintained upon freeze-drying even in the presence of 6% *v*/*v* of ethanol and a unimodal particle distribution (Figure 5). The result is acceptable also considering that, in the case of parental formulations, filtration of the final product is required. Despite the very few reports dealing with the effects of secondary drying on the product stability, it can be assumed that the slow ramp of the shelf temperature toward secondary drying (0.1 K min^−1^) would avoid jeopardization of product properties during the desorption of residual moisture content, which is fairly high in amorphous excipients [25].

Indeed, it can be hypothesized that, upon desorption, the product temperature slowly increased without exceeding T_m_ of DPPC.

## 4. Conclusions

A deep knowledge of the formulation and process parameters and the intricated relationship among variables is required to effectively protect liposomes during freeze-drying. The situation gets even more complex when ethanol is concomitantly present in the formulation. Indeed, a solvent can impact both the freezing and the sublimation steps. As demonstrated in this report, ethanol causes a massive deviation of T_g_′. The relatively low vapour pressure of ethanol, which might slow down the sublimation phase, can be counterweighted by the variation of temperature during the primary drying and the heating ramp rate towards secondary drying. Regarding the design of formulation, the combination of two excipients approved for parenteral administration was effective in preserving liposome structure upon reconstitution of final freeze-dried products with adequate morphology, irrespective of the ethanol content.

In conclusion, although further studies on a wider range of liposomal formulations are required, this work reports a proof of concept on lyophilization of DPPC liposomes prepared by ethanol injection, avoiding the intermediate step of solvent evaporation and, thus, decreasing the time and the costs of the overall production process of a pharmaceutical form. Furthermore, it confirms that MD simulations can be a useful tool to rationalize the selection of protectant(s) when freeze-drying complex formulations.

## Figures and Tables

**Table 1:** Schematization of process parameters of lyo-cycles.

Lyo-Cycle	Freezing	Primary Drying	Secondary Drying
A	−40 °C 8 h	−40 °C	25 °C
		0.2 mbar	0.2 mbar
		48 h	10 h
B	−40 °C 8 h	−48 °C	25 °C
		0.2 mbar	0.2 mbar
		48 h	10 h
C	−48 °C 8 h	−48 °C for 12 h	25 °C
		−40 °C for 13 h	0.1 mbar
		0.1 mbar	6 h

**Table 2:** Hydrodynamic diameter (D_H_) and polydispersity index (PdI) of liposomes prepared by thin-film hydration methods after thawing in the presence of different excipients or their combinations: Disaccharide and DPPC were in a 5:1 molar ratio. PVP was added in different percentages, m/v.

Form ID	Composition		Before Freezing		After Thawing	
	Disaccharide	PVP	D_h_ (nm)	PDI	D_h_ (nm)	PDI
1	Trehalose	-	170 ± 1	0.04 ± 0.02	169 ± 9	0.14 ± 0.06
2	Sucrose	-	171 ± 2	0.05 ± 0.03	200 ± 18	0.29 ± 0.03
3	-	0.50	172 ± 1	0.03 ± 0.01	- *	- *
4	-	0.75	171 ± 3	0.03 ± 0.02	- *	- *
5	Trehalose	0.50	179 ± 2	0.10 ± 0.02	205 ± 9	0.15 ± 0.01
6	Trehalose	0.75	176 ± 2	0.12 ± 0.01	>400	>0.4
7	Sucrose	0.50	180 ± 3	0.11 ± 0.01	209 ± 0	0.19 ± 0.04

* too-dispersed samples due to the formation of large aggregates.

**Table 3:** Thermotropic behavior of DPPC in the lamellar phase rehydrated in the presence of water or a solution of trehalose (5:1 molar ratio), PVP, or a mixture thereof: The same excipients were also added to the rehydrated lamellar phase.

Inner Phase (Liposome Core)	Outer Phase (Dispersant)	T_p_			T_m_		
		∆H (J/g)	T_onset_ (°C)	T_peak_ (°C)	∆H (J/g)	T_onset_ (°C)	T_peak_ (°C)
-	-	3.21 ± 0.96	34.16 ± 0.40	35.39 ± 0.19	55.54 ± 0.33	41.39 ± 0.02	41.82 ± 0.03
Trehalose	Trehalose	4.88 ± 0.23	33.94 ± 0.47	35.69 ± 0.47	62.55 ± 1.41	40.89 ± 0.32	41.59 ± 0.16
PVP	PVP	-	-	-	36.36 ± 0.44	41.51 ± 0.08	41.95 ± 0.09
Water	PVP/trehalose	1.94 ± 0.21	35.23 ± 0.40	36.26 ± 0.04	40.92 ± 0.46	41.40 ± 0.02	41.96 ± 0.15
Trehalose	PVP/trehalose	4.63 ± 0.06	35.01 ± 0.22	36.47 ± 0.31	36.48 ± 1.86	41.59 ± 0.04	41.90 ± 0.19
PVP/trehalose	PVP/trehalose	4.65 ± 0.79	35.83 ± 0.15	37.35 ± 0.19	42.69 ± 0.97	41.87 ± 0.10	42.12 ± 0.12

**Table 4:** Glass transition of the freeze-concentrated (T_g_′) solution of trehalose, PVP, and the combination thereof in the presence of different ethanol contents.

% Ethanol	T_g_′ (°C)		
	Trehalose	PVP	Mixture
0	−28.53 ± 1.44	−28.32 ± 0.68	−27.63 ± 0.06
0.1	−29.35 ± 1.52	−32.48 ± 0.48	−30.17 ± 1.10
1	−35.42 ± 1.53	−47.98 ± 3.16	−45.04 ± 0.86
5	−45.47 ± 1.61	- ^1^	- ^1^

^1^ below the limit of detection of the instrument.

**Table 5:** Dynamic light scattering (DLS) results on liposomes before freeze-drying and after rehydration: All samples were freeze-dried according to method A.

Ethanol (%)	Trehalose: DPPC (mol/mol)	PVP (%, m/v)	Pre-Lyophilization		Post-Lyophilization	
			D_H_ (nm)	PDI	D_H_ (nm)	PDI
0	5:1	-	170 ± 1	0.04 ± 0.02	286 ± 24	0.38 ± 0.03
	5:1	0.5	185 ± 1	0.12 ± 0.03	261 ± 4	0.20 ± 0.02
0.1	5:1	-	112 ± 0	0.14 ± 0.12	213 ± 14	0.39 ± 0.00
	5:1	0.5	173 ± 2	0.28 ± 0.00	194 ± 16	0.30 ± 0.01
1.0	5:1	-	118 ± 1	0.16 ± 0.00	216 ± 51	0.48 ± 0.20
	5:1	0.5	125 ± 0	0.22 ± 0.00	266 ± 22	0.39 ± 0.06
6.0	5:1	-	158 ± 2	0.25 ± 0.01	512 ± 133	0.53 ± 0.14
	5:1	0.5	158 ± 2	0.23 ± 0.02	484 ± 368	0.48 ± 0.26

## Supplementary Materials

The following are available online at https://www.mdpi.com/1999-4923/12/6/530/s1, Table S1: Composition and experimental setup to prepare liposomes by ethanol injection, Table S2: Reproducibility of the main physicochemical properties of formulation prepared by ethanol injection (E1–E11) or the thin-film hydration method (H1–H4): As expected, liposomes prepared by thin-film hydration method presented a higher diameter and lower PDI than those prepared by ethanol injection, Figure S1: Thermotropic behavior of DPPC (black line) in the presence of trehalose (red line), PVP (blue line), or a mixture thereof (green line), Figure S2: DSC thermograms of solution of trehalose in the presence of 0.1 (black line), 1% (red line), and 6% (blue line) ethanol content, Figure S3: DSC thermograms of trehalose before freeze-drying (black line) and trehalose/PVP after freeze-drying (red line), Figure S4: DLS of reconstituted liposomes after freeze-drying a dispersion in the presence of trehalose and 6% ethanol. The data are represented as size distribution by intensity.
