# Supplementary material for: Preserving the Integrity of Liposomes Prepared by Ethanol Injection upon Freeze-Drying: Insights from Combined Molecular Dynamics Simulations and Experimental Data
Source: Pharmaceutics. 2020 Jun 9;12(6):530. doi: 10.3390/pharmaceutics12060530 (PMC7356173; doi:10.3390/pharmaceutics12060530)
Supplement: Supplementary file 1 [file pharmaceutics-12-00530-s001.pdf]

pharmaceutics

MDPI

Preserving the Integrity of Liposomes Prepared by Ethanol Injection Upon Freeze-Drying: Insights from Combined Molecular Dynamics Simulations and Experimental Data

Silvia Franzè, Francesca Selmin, Paolo Rocco, Giuseppe Colombo, Antonella Casiraghi and Francesco Cilurzo

Table 1. Composition and experimental set-up to prepare liposomes by ethanol injection.

Form	Total concentration in EtOH (mM)	EtOH/water ratio (%)		Injection rate (mL/min)	Stirring rate (rpm)	Temperature (°C)	Size (nm)	PDI	ζ (mV)
1	50	16.7	83.3	1.5	400	25	-*	-*	-*
2	25	20	80	1.0	250	25	>950	-	n.d.
3	25	11.1	89.9	1.0	300	25	>950	-	n.d.
4	25	5.9	94.1	1.0	300	25	277±25	0.24±0.00	-9.53±0.39
5	25	5.9	94.1	1.0	300	55	131±1	0.14±0.01	-8.00±1.12

* visible aggregates.

Table S2. Reproducibility of main physico-chemical properties of formulation prepared by ethanol injection (E1–E11) or the thin film hydration method (H1-H4). As expected, liposomes prepared by thin film hydration method presented a higher diameter and lower PDI than those prepared by ethanol injection.

Batch ID	Diameter (nm)	PdI	ζ (mV)
E1	131±1	0.14±0.01	-8.0±1.1
E2	132±1	0.17±0.01	-9.8±0.7
E3	137±1	0.14±0.02	-10.5±0.4
E4	151±1	0.15±0.02	-9.6± 1.0
E5	160±1	0.08±0.03	-16.2±0.9
E6	142±1	0.13±0.04	-14.5± 0.3
E7	122±0	0.17±0.01	-8.9±0.5
H1	172±1	0.04±0.02	-10.8±0.5
H2	178±1	0.05±0.03	-15.0±0.6
H3	178±2	0.08± 0.01	-5.9±0.5
H4	172±0	0.06±0.02	-10.3±0.5

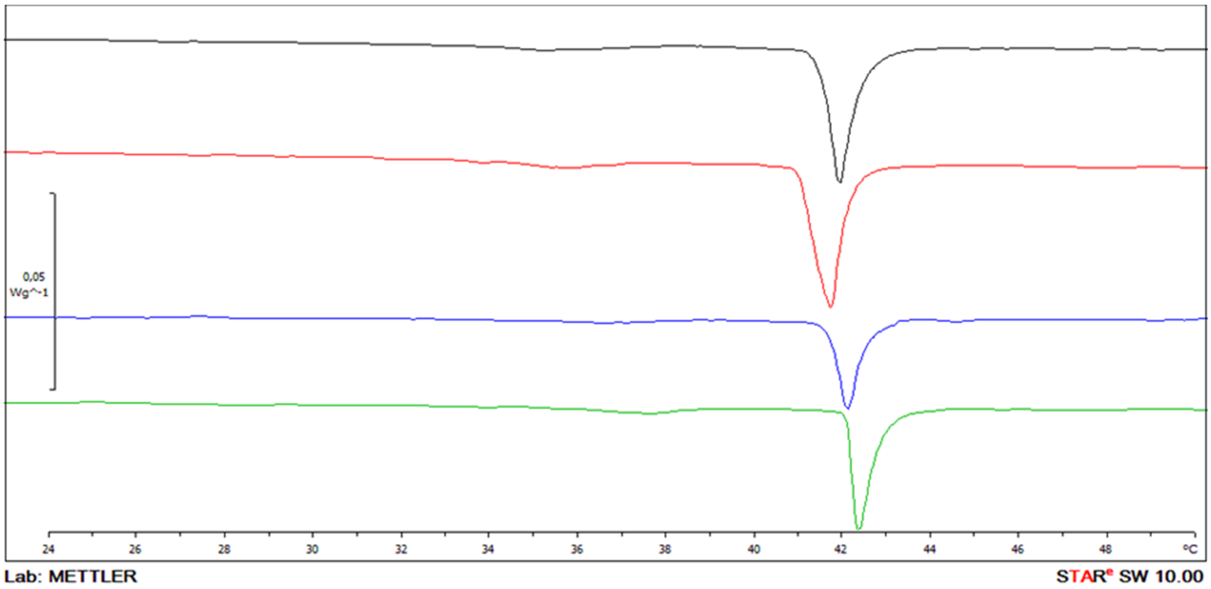

Figure S1. Thermotropic behavior of DPPC (black line) in presence of trehalose (red line), or PVP (blue line) or a mixture thereof (green line).

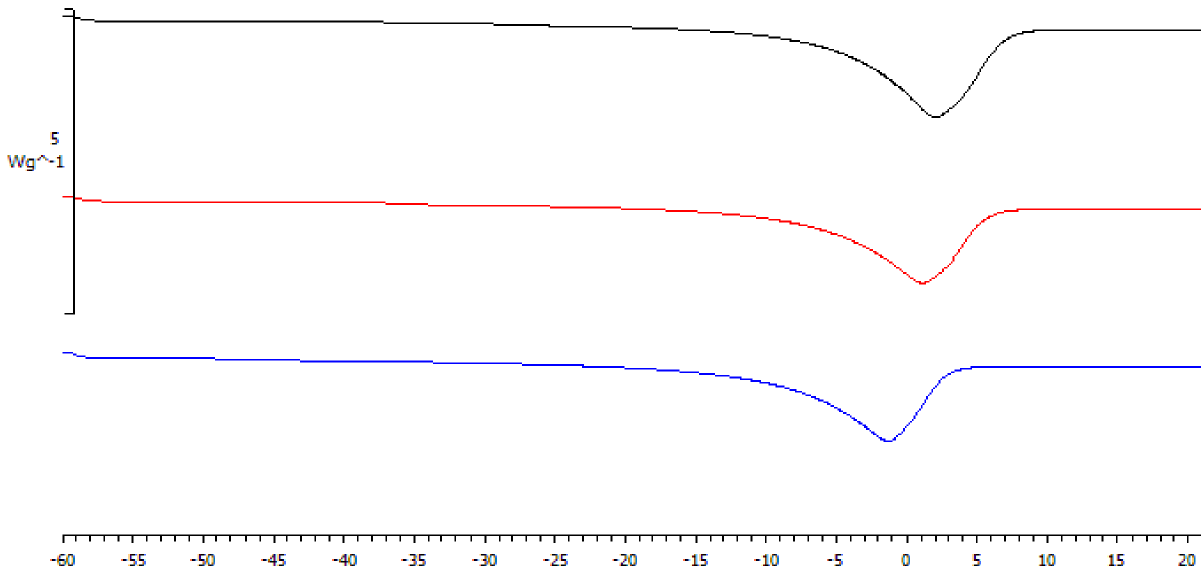

Figure S2. Reports DSC thermograms of solution of trehalose in presence of 0.1 (black line), 1% (red line) and 6% (blue line) ethanol content. Two thermal events are observed during the heating phase: first is glass transition temperature (T_g') of maximally freeze concentrated solution and second is melting endotherm associated with melting of frozen solution. The DSC measurement of glass transition temperature (T_g') of maximally freeze concentrated solution shows a change in the baseline heat flow signal resulting from a increase in heat capacity associated with glass transition during heating ramp.

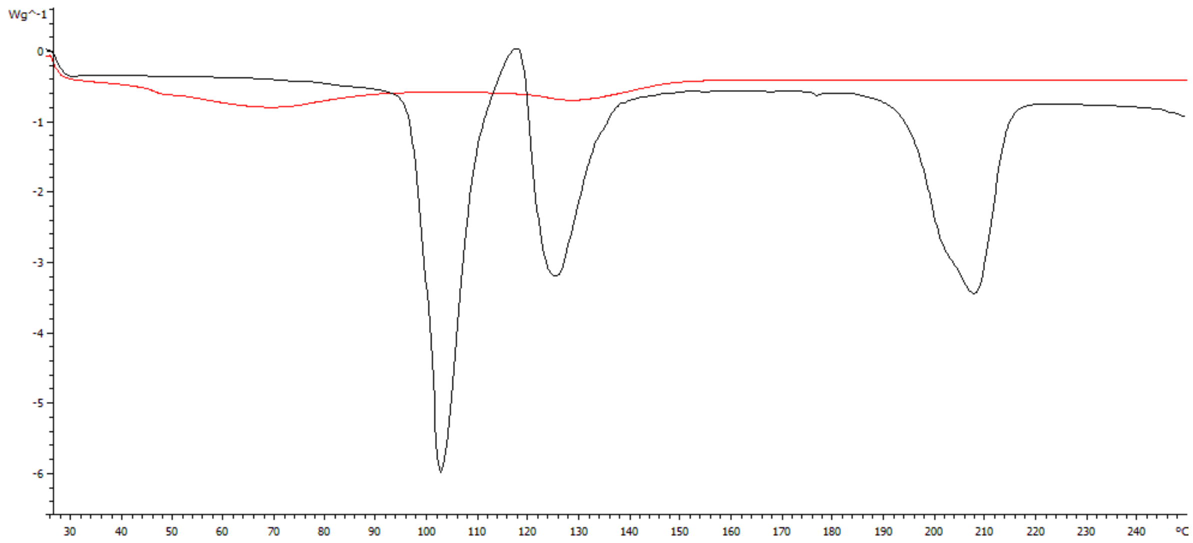

Figure S3. DSC thermograms of trehalose dihydrate before freeze-drying (black line) and trehalose/PVP after freeze-drying (red line).

In the case of trehalose (black line), these events were detected:

- a sharp endotherm peak at about 100 °C due to the loss of 1 molecule of water,
- an exothermic peak due to the arrangement,
- a broader endotherm transition at 122 °C due to the loss of 1 molecule of water,
- sharp, shouldered endotherm at 211 °C, attributed to the melting of the anhydrous compound.

The raw PVP K12 presented a T_g at about 105 °C(data not shown).

The DSC trace related to the threalose/PVP blend (red line) is typical of an amorphous product where endothermic peak of trehalose and the T^g of PVP are lacking suggesting the formation of an intimate blend in agreement to the MD simulations. Indeed, the only significant detectable event is the a T_g at about 46°C appeared. This value was lower than those of PVP or amorphous trehalose in presence of small amount of water (~90°C, OS McGarvey, VL Kett, and DQM Craig. Crystallization of α-α trehalose from the amorphous state. J. Phys. Chem. B, Vol. 107, No. 27, 2003 6617) in agreement with the hypothesis of formation of a solid solution. However, this topic, which require further work, is beside the aim of the actual study.

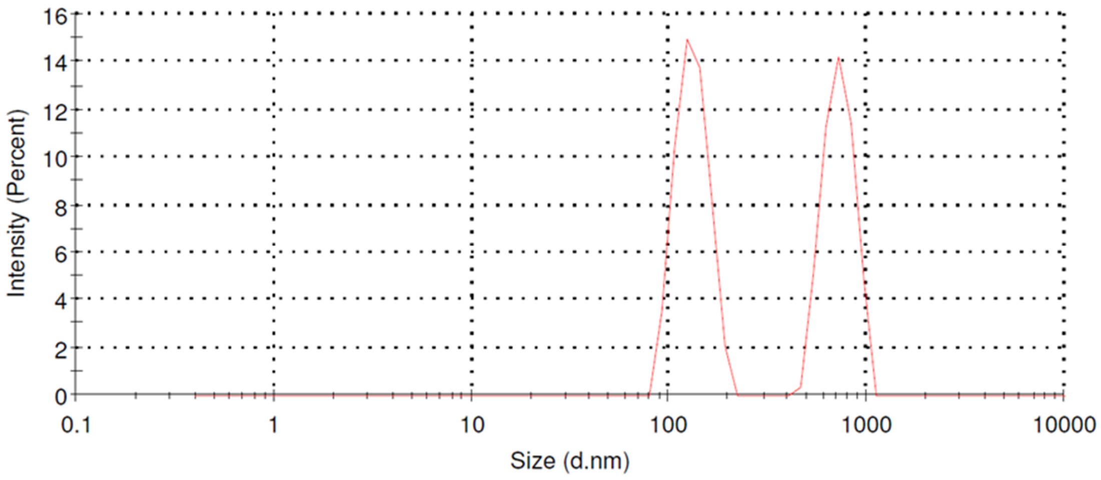

Figure S4. DLS of reconstituted liposomes after freeze-drying a dispersion in presence of trehalose and 6% ethanol. The data are represented as size distribution by intensity.
